# Zoanthamine Alkaloids from the Zoantharian *Zoanthus* cf. *pulchellus* and Their Effects in Neuroinflammation

**DOI:** 10.3390/md16070242

**Published:** 2018-07-20

**Authors:** Paul O. Guillen, Sandra Gegunde, Karla B. Jaramillo, Amparo Alfonso, Kevin Calabro, Eva Alonso, Jenny Rodriguez, Luis M. Botana, Olivier P. Thomas

**Affiliations:** 1 ESPOL Escuela Superior Politécnica del Litoral, ESPOL, Centro Nacional de Acuacultura e Investigaciones Marinas, Campus Gustavo Galindo km. 30.5 vía Perimetral, P.O. Box 09-01-5863 Guayaquil, Ecuador; P.GUILLENMENA1@nuigalway.ie (P.O.G.); K.JARAMILLOAGUILAR1@nuigalway.ie (K.B.J.); jenrodri@espol.edu.ec (J.R.); 2 Marine Biodiscovery, School of Chemistry and Ryan Institute, National University of Ireland Galway (NUI Galway), University Road, H91 TK33 Galway, Ireland; kevin.calabro@nuigalway.ie; 3 Departamento de Farmacología, Facultad de Veterinaria, Universidade de Santiago de Compostela, 27002 Lugo, Spain; sandra.gegunde@rai.usc.es (S.G.); amparo.alfonso@usc.es (A.A.); eva.alonso@usc.es (E.A.); 4 Zoology, School of Natural Sciences and Ryan Institute, National University of Ireland Galway (NUI Galway), University Road, H91 TK33 Galway, Ireland

**Keywords:** zoantharia, Tropical Eastern Pacific, *Zoanthus pulchellus*, zoanthamine, inflammation

## Abstract

Two new zoanthamine alkaloids, namely 3-acetoxynorzoanthamine (**1**) and 3-acetoxyzoanthamine (**2**), have been isolated from the zoantharian *Zoanthus* cf. *pulchellus* collected off the coast of the Santa Elena Peninsula, Ecuador, together with three known derivatives: zoanthamine, norzoanthamine, and 3-hydroxynorzoanthamine. The chemical structures of **1** and **2** were determined by interpretation of their 1D and 2D NMR data and comparison with literature data. This is the first report of zoanthamine-type alkaloids from *Zoanthus* cf. *pulchellus* collected in the Tropical Eastern Pacific. The neuroinflammatory activity of all the isolated compounds was evaluated in microglia BV-2 cells and high inhibitory effects were observed in reactive oxygen species (ROS) and nitric oxide (NO) generation.

## 1. Introduction

Zoanthamines are a bioactive family of marine alkaloids featuring a unique chemical architecture of fused cycles culminating in an unusual azepane ring. They have been isolated essentially from marine zoantharians, particularly from the genus *Zoanthus.* The first alkaloid of this group was isolated in 1984 from an unidentified species of *Zoanthus*, collected off the coast of India by Faulkner et al. [1]. Following this first description, several studies on the chemical diversity of species of the genus *Zoanthus* have led to the discovery of additional zoanthamine-type alkaloids, including zoanthenamine, zoanthenamide [2], norzoanthamine, oxyzoanthamine, norzoanthaminone, cyclozoanthamine, epinorzoanthamine [3], zoanthaminone [4], zoaramine [5], kuroshines [6], epioxyzoanthamine [7], zoanthenol [8], hydroxylated zoanthamines and norzoanthamines [9], and two halogenated zoanthamines [10]. This interesting family of alkaloids has been structurally classified in two different groups based on the presence of a methyl at C-19 (Type I) or its absence (Type II), also called norzoanthamines [10]. Due to the structural complexity of these natural products, the first total synthesis of norzoanthamine was accomplished by Miyashita et al. in 2004 [11], who also synthesized other analogues [12,13]. Other research groups are now addressing this synthetic challenge through alternative approaches [14,15,16]. Up to date, 38 zoanthamine-type alkaloids have been reported from zoantharian species essentially inhabiting the Central Indo-Pacific and these polycyclic alkaloids seem to be chemical markers of zoantharians from the genus *Zoanthus*. In addition, some members of this family have displayed a wide range of biological activities against P388 murine leukemia cells [3] as well as anti-osteoporosis, anti-inflammatory, and anti-bacterial activity, and have also been found to inhibit human platelet aggregation [9,17]. The most promising therapeutic application is associated with norzoanthamine in the treatment of osteoporosis, as it inhibits interleukin-6, a primary mediator of bone resorption. Furthermore, an interesting study by Tachibana et al. suggested that the principal function of norzoanthamine in *Zoanthus* sp. is collagen strengthening [18].

**Figure 1:**
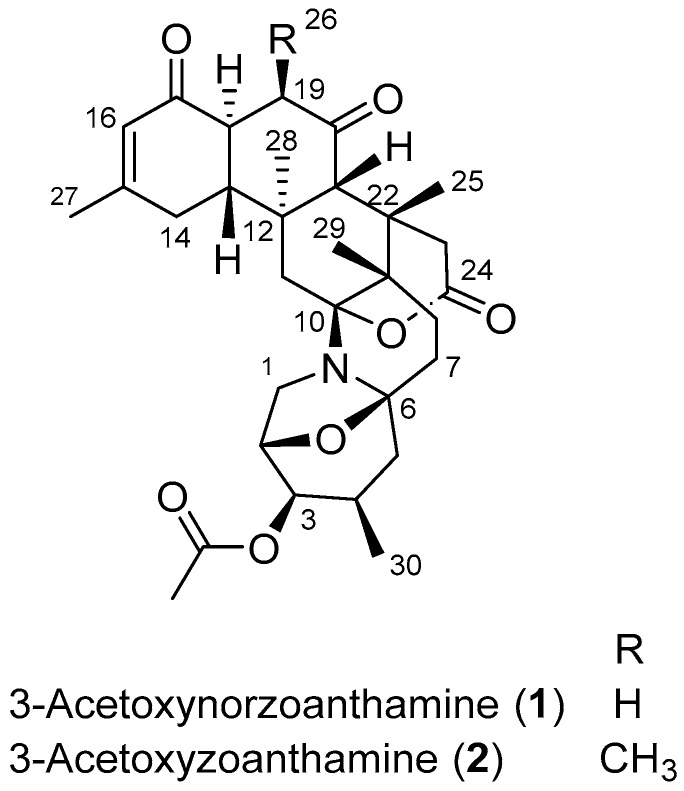
Structures of 3-acetoxynorzoanthamine (**1**) and 3-acetoxyzoanthamine (**2**), isolated from *Zoanthus* cf. *pulchellus.*

In our continuous investigation of the bio- and chemodiversity of marine invertebrates present in the understudied Marine Protected Area El Pelado, Santa Elena, Ecuador, located in the Tropical Eastern Pacific [19,20], we came across a massive substrate cover of the intertidal region by undescribed fluorescent green zoantharians. A first taxonomic assessment of these zoantharian species led to the identification of the main species as being closely related to *Zoanthus* cf. *pulchellus*, previously described in the Caribbean [21]. No chemical study of this species has been reported so far, and our first chemical screening by UHPLC-HRMS revealed unknown masses related to the zoanthamine family as major compounds of the extract. In this paper, we describe the isolation and structure elucidation of two new zoanthamine alkaloids, namely 3-acetoxynorzoanthamine (**1**) and 3-acetoxyzoanthamine (**2**) (Figure 1), along with the known zoanthamine [1], norzoanthamine [3], and 3-hydroxynorzoanthamine [8] from the Eastern Pacific zoantharian *Zoanthus* cf. *pulchellus*, as well as their biological activity in cellular pathways related to oxidative stress and neuroinflammation.

## 2. Results

Colonies of the zoantharian *Zoanthus* cf. *pulchellus* were collected by hand in the intertidal coast of San Pedro, Santa Elena, Ecuador. The sample was freeze-dried and extracted with a mixture of solvents CH_3_OH:CH_2_Cl_2_ (*v*/*v*; 1:1). The extract was then fractionated through reversed-phase C18 Vacuum Liquid Chromatography (VLC) using a mixture of solvents of decreasing polarity. The aqueous methanolic fractions were analyzed by UPLC-DAD-ELSD, combined, and then subjected to semipreparative RP-HPLC using a C18 column to yield two new zoanthamine-type alkaloids: 3-acetoxynorzoanthamine (**1**) and 3-acetoxyzoanthamine (**2**), along with the known zoanthamine [1], norzoanthamine [18], and 3-hydroxynorzoanthamine [8].

Compound **1** was obtained as a brown amorphous powder and (+)-HRESIMS analyses revealed a major molecular peak at *m*/*z* 540.2956 [M + H]^+^, consistent with the molecular formula C_31_H_41_NO_7_ for the neutral molecule_._ A preliminary inspection of the ^1^H and ^13^C NMR data revealed characteristic signals of the zoanthamine family, as already speculated on the basis of the HRMS data: an olefinic proton at *δ*_H_ 5.90 (H-16) along with four methyl singlets at *δ*_H_ 0.97 (H-28), 0.99 (H-25), 1.15 (H-29), and 2.00 (H-27), and a methyl doublet at *δ*_H_ 0.87 (H-30) together with two ketone signals at *δ*_C_ 198.5 (C-17) and *δ*_C_ 209.0 (C-20), one ester signal at *δ*_C_ 172.3 (C-24), and two olefinic carbons at *δ*_C_ 125.6 (C-16) and 160.0 (C-15) (Table 1). The absence of a second doublet of a methyl present in zoanthamines was indicative of a loss of the methyl CH_3_-26 at C-19; therefore, the compound belonged to the norzoanthamine-type. Unlike most studies on norzoanthamines, in order to make the NMR table more homogeneous, we decided to keep the numbering of the zoanthamines especially for the methyls 27, 28, 29, and 30. Comparing with analogues of this type, we observed the presence of an additional methyl singlet signal at *δ*_H_ 2.11 corresponding to an acetyl moiety (Table 1). The presence of the acetyl group on an oxygen at C-3 was evidenced by the deshielding of the signal corresponding to the methine H-3 with *δ*_H_ 4.62 and key H-3/C-1′ and H_3_-2′/C-1′ HMBC correlations.

We then addressed the question of the relative configurations of the different chiral centers. To the best of our knowledge, this is the first occurrence of an acetoxy group at position C-3 for zoanthamines; however, other analogues oxygenated at this position have already been described. First, 3-hydroxynorzoanthamine was isolated from an undescribed species of *Zoanthus* from the Canary Islands in the Atlantic Ocean [8]. Later, kuroshines C and F as well as 3*β*-hydroxyzoanthenamide also possess an hydroxyl group at this position [6]. All these four derivatives were shown to have a hydroxyl group on the *β*-side of the polycyclic compound and this position was deduced from nOes between H-3 and other protons of the azepane ring. In our case, and because both H-3/H-4a and H-3/H-4b coupling constant values were not fully conclusive, we relied on the key H-3/H-1b nOe correlation to place H-3 on the opposite side of the bridged oxygen (*α*-side). Subsequently, the acetoxy group was located on the *β*-side like for the other four 3-hydroxylated analogues. The very low coupling constant values of H-3 with H-2 and H-4 were similar to those observed for all 3-hydroxylated compounds and in perfect agreement with this relative configuration. Additionally, a previous study by Uemura et al. assigned the absolute configuration of norzoanthamine as 2*R*, 4*S*, 6*S*, 9*S*, 10*R*, 12*R*, 13*R*, 18*S*, 21*S*, and 22*S* and suggest the same absolute configuration for all norzoanthamine-type alkaloids [22]. In our case, the positive specific rotation obtained for **1** was in accordance with that obtained for 3-hydroxyzoanthamine and therefore confirmed the same absolute configuration [8].

Compound **2** was isolated as an amorphous yellowish powder and the molecular formula C_32_H_43_NO_7_ was deduced from HRESIMS revealing a major peak at *m*/*z* 554.3115 [M + H]^+^; therefore, **2** is an homologue of **1**. A quick inspection of the ^1^H NMR spectrum evidenced the presence of the acetoxy group at C-3 as in **1**. An additional methyl signal at *δ*_H_ 1.17 (d, *J* = 7.0 Hz, H_3_-26) suggested that **2** is a member of the zoanthamine-type alkaloids. The presence of the methyl at C-19 was confirmed by the key H-19/C-26 and H_3_-26/C-18/C-19 HMBC correlations. The *β*-position of the methyl 26 was then inferred from the coupling constant value *J*_H-18/H-19_ of 6.0 Hz, reminiscent of an axial/equatorial coupling. Because H-18 is placed in an axial position, H-19 should be placed in an equatorial position; therefore, the methyl 26 occupies the corresponding axial *β*-position at C-19. The *β*-position of the acetoxy at C-3 was deduced from the same coupling constant values of H-3 as for **1,** and the absolute configuration was supposed to be the same as that of **1**, again because of similar positive specific rotations.

**Figure 2:**
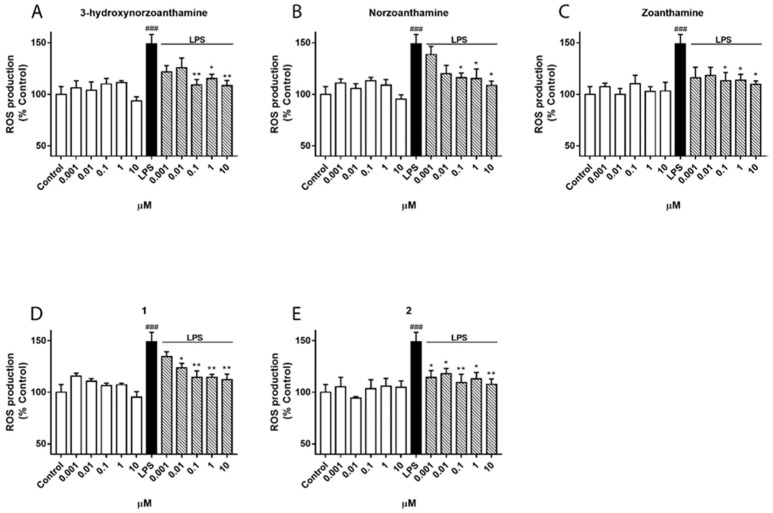
Effect of zoanthamines on intracellular reactive oxygen species (ROS) production in microglia BV-2 cell line. Cells were pre-treated with 3-hydroxynorzoanthamine (**A**); norzoanthamine (**B**); zoanthamine (**C**); **1** (**D**); and **2** (**E**) at different concentrations (0.001, 0.01, 0.1, 1, and 10 µM) 1 h and then stimulated with lipolysaccharide (LPS) (1 µg/mL) for 24 h. ROS production is presented as a percentage of cells control, being the result of mean fluorescence intensity ± SEM of three independent experiments. The values are shown as the difference between cells treated with LPS alone versus cells treated with zoanthamines in presence of LPS by ANOVA followed by *post hoc* Dunnett’s test. * *p* < 0.05 and ** *p* < 0.01, and LPS-treated cells versus control cells ### *p* < 0.001.

**Figure 3:**
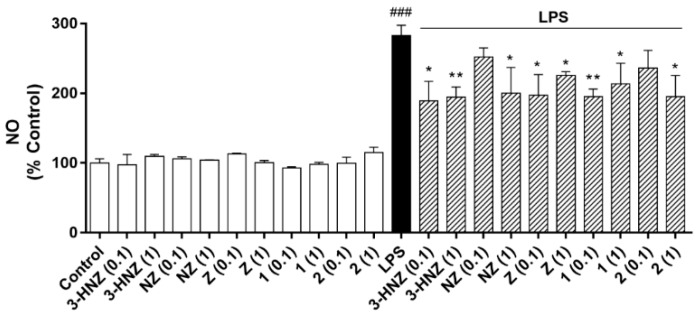
Effect of zoanthamines on nitric oxide (NO) production in BV-2 microglia cell line. Cells were pre-treated with 3-hydroxynorzoanthamine (3-HNZ), norzoanthamine (NZ), zoanthamine (Z), **1**, and **2** (0.1 or 1 µM) for 1 h and then stimulated with lipolysaccharide (LPS) (500 ng/mL) for 24 h. The values are presented in percentage of cells control, being the result of mean ± SEM of a minimum of three independent experiments. The cells treated only with LPS were compared to cells treated with compounds in presence of LPS by ANOVA followed by *post hoc* Dunnett’s test. * *p* < 0.05 and ** *p* < 0.01, and LPS-treated cells versus control cells ### *p* < 0.001.

The compounds were tested for biological activity in the BV-2 microglia cell line, a cellular model often used in neuroinflammation studies. The first step was to determine the effect of compounds on cell viability. Five concentrations (from 0.001 to 10 µM) were investigated and after 24 h of incubation no effects on cell viability were observed, which suggested non-toxic compounds. Microglia-mediated inflammation is known to produce reactive oxygen species (ROS) and release nitric oxide (NO), and thus induce oxidative damage [23]. Therefore, zoanthamines were checked as modulators within these processes. BV-2 cells were activated with lipolysaccharide (LPS) to simulate neuroinflammatory conditions. As shown in Figure 2, when cells were pre-treated with the same concentrations of compounds for 1 h and then incubated for 24 h with LPS (500 ng/mL), a significant reduction in ROS production was observed. As expected, the stimulation of BV-2 cells with LPS significantly increased the ROS production, 50% (*p* < 0.001), while the compounds alone did not induce any effect. However, when cells were pre-treated with norzoanthamine and **1**, a dose-dependent inhibitory effect was observed, while 3-hydroxynorzoanthamine, zoanthamine, or **2** were effective at all concentrations tested, with **2** being the most potent ROS inhibitor. From these results, 0.1 and 1 µM were chosen to investigate the effect on NO release (Figure 3). Zoanthamine alkaloids alone did not produce any effect on NO production, while LPS treatment increased it by three times. In the presence of this family of compounds, NO release was significantly inhibited. The anti-inflammatory effect of zoanthamines was previously investigated in neutrophils [10]. From our results in the BV-2 cellular model, zoanthamine and derivatives show effective properties as protective drugs in neuroinflammation processes.

## 3. Discussion

The isolation of two 3-acetoxy derivatives of zoanthamine and norzoanthamine in *Zoanthus* cf. *pulchellus* strengthens the hypothesis that zoanthamines are markers of the genus *Zoanthus*. However, another species identified as *Zoanthus* cf. *sociatus* was found in the same area and did not present any zoanthamine derivatives [21]. Nevertheless, even if these compounds should not be considered as taxonomic markers of the genus *Zoanthus*, they are clear and characteristic features of some species of *Zoanthus* and could facilitate a more precise classification of this group.

Interestingly, we first ran the NMR analyses of **1** in a different solvent, CD_3_OD, and observed clear changes for the signals surrounding the nitrogen atom. Especially, the signals corresponding to H-11 disappeared. This observation reinforced the conclusions on zoanthamine analogues reached by the group of Norte [8]. In a highly polar and protic solvent, the opening of the lactone ring would give rise to an iminium ion at C-11 in equilibrium with its enamine base that can be trapped by exchangeable deuterium atoms provided by the protic deuterated solvent. This behavior signals the high reactivity of this family of compounds at this particular position.

Because these compounds were isolated after a purification step involving acetic acid in the eluent of the HPLC, we wanted to ascertain the presence of these compounds in the collected specimen. For this purpose, we inspected the chemical profiles obtained before any contact with acetic acid and were able to observe the masses corresponding to the new compounds **1** and **2**. These analyses rule out the possibility of a transformation during the purification process.

Finally, the activity observed for all compounds highlights the potential of zoanthamine derivatives as new ROS and NO modulators in neuronal processes, and we will continue our efforts in the study of their mode of action in neuroinflammatory related diseases.

## 4. Materials and Methods

### 4.1. General Experimental Procedures

Optical rotation measurements were obtained at the sodium D line (589.3 nm) with a 10-cm cell at 20 °C on a UniPol L1000 polarimeter (Schmidt + Haensch, Berlin, Germany). The UV measurements were obtained on a Cary 300 UV-Visible spectrophotometer (Agilent, Santa-Clara, CA, USA). NMR spectra were recorded on a Inova 500 MHz spectrometer (500 and 125 MHz for ^1^H and ^13^C, respectively) (Varian, Palo Alto, CA, USA), and signals were referenced in ppm to the residual solvent signals (CDCl_3_, at *δ*_H_ 7.26 and *δ*_C_ 77.16 ppm). HRESIMS data were obtained with a UHPLC-qTOF 6540 mass spectrometer (Agilent, Santa Clara, CA, USA). Purification was carried out on a HPLC equipped with a PU4087 pump (JASCO, Tokyo, Japan) and a UV4070 UV/Vis detector (JASCO, UV, Tokyo, Japan).

### 4.2. Biological Material

Specimens of *Zoanthus* cf. *pulchellus* were collected by hand on rocks of the shoreline of San Pedro located in the Santa Elena Peninsula, Ecuador. A sample with a voucher 161125SP-01 is stored at CENAIM-ESPOL (San Pedro, Santa Elena, Ecuador). This species has been previously identified using morphological and molecular data [21].

### 4.3. Extraction and Isolation

The freeze-dried sample of *Z.* cf. *pulchellus* (200 g) was extracted with a mixture of solvents DCM/MeOH (1:1) three times (500 mL) at room temperature. The collected extract was concentrated under reduced pressure to obtain the extract (10 g). The extract was subjected to C18 reversed-phase VLC (LiChroprep^®^ (Merck KGaA, Darmstadt, Germany) RP-18, 40–63 µm, 1:25 ratio for the weight of C18 used, funnel of 10 cm × 10 cm) using a mixture of solvents of decreasing polarity (1) H_2_O; (2) H_2_O/MeOH (1:1); (3) H_2_O/MeOH (1:3); (4) MeOH; (5) MeOH/DCM (3:1); (6) MeOH/DCM (1:1); and (7) DCM using 500 mL of each solvent. The aqueous-methanolic fraction F3 was purified by reversed-phase HPLC (Ultra AQ C18, 10 × 250 mm, 5 µm) using an isocratic method CH_3_CN:H_2_O:Acetic acid (30:70:0.1) as a mobile phase with a flow rate of 3 mL/min with detection at *λ* 254 nm for 20 min yielding compound **1** (52.7 mg) and the known compounds norzoanthamine (6.3 mg) [3] and zoanthamine (6.6 mg) [1]. The methanolic fraction F4 was purified by reversed-phase HPLC (Ultra AQ C18, 10 × 250 mm, 5 µm) using the following mobile phases: (A) CH_3_CN/Acetic acid 0.1%; (B) H_2_O/Acetic acid 0.1%; starting with an isocratic 0–25 min with A 22, B 78; linear gradient for 25–30 min until A 100; then isocratic for 30–60 min at a flow rate of 3 mL/min with UV detection at *λ* 254 nm to yield compounds **2** (12.3 mg) and the known 3-hydroxynorzoanthamine (2.7 mg) [8].

### 4.4. 3-Acetoxynorzoanthamine (***1***)

Amorphous yellow powder; ${\left[\alpha \right]}_{D}^{20}$ +10 (*c* 0.45, CH_3_OH); UV (CH_3_OH) *λ*_max_ (log *ε*) 237 (4.1) nm; ^1^H NMR and ^13^C NMR data see Table 1; HRESIMS (+) *m*/*z* [M + H]^+^ 540.2956 (calc. for C_31_H_42_NO_7_ 540.2956Δ + 0.0 ppm) (Spectra in the Supplementary Materials).

### 4.5. 3-Acetoxyzoanthamine (***2***)

Amorphous yellowish powder; ${\left[\alpha \right]}_{D}^{20}$ + 6.7 (*c* 0.12, CH_3_OH); UV (CH_3_OH) *λ*_max_ (log ε) 238 (4.0) nm; ^1^H NMR and ^13^C NMR data see Table 1; HRESIMS (+) *m*/*z* [M + H]^+^ 554.3115 (calc. for C_32_H_44_NO_7_ 554.3112Δ + 0.5 ppm) (Spectra in the Supplementary Materials).

### 4.6. Biological Assays

#### 4.6.1. Cell Culture

The microglia BV-2 cell line was obtained from InterLab Cell Line Collection (ICLC) (Genova, Italy), number ATL03001. Cells were maintained in Roswell Park Memorial Institute Medium (RPMI) supplemented with 10% fetal bovine serum (FBS), penicillin (100 U/mL), and 100 µg/mL streptomycin at 37 °C in a humidified atmosphere of 5% CO_2_ and 95% air. Cells were dissociated twice a week using 0.05% trypsin/EDTA.

#### 4.6.2. Cell Viability

The 3-(4,5-dimethyl thiazol-2-yl)-2,5-diphenyl tetrazolium bromide (MTT) assay was used to analyzed cell viability as previously described [24]. Briefly, the microglia BV-2 cell line was grown in a 96-well plate at a density of 4 × 10^4^ cells per well. Cells were exposed to different compounds concentration (0.001, 0.01, 0.1, 1 and 10 µM) for 24 h. Then, cells were rinsed and incubated with MTT (500 µg/mL) diluted in a saline buffer for 1 h at 37 °C. The resulting formazan crystals were dissolved with 5% sodium dodecyl sulfate, (SDS) and the absorbance values were obtained using a spectrophotometer plate reader (595 nm). Saponin was used for cellular death control and its absorbance was substrate from the other data.

#### 4.6.3. Measurement of Intracellular ROS Production

The intracellular ROS levels in microglia activation were performed using 7′,2′-dichlorofluorescein diacetate (DCFH-DA), as previously described [25]. Cells were pre-treated with different compounds concentration (0.001, 0.01, 0.1, 1, and 10 µM) 1 h prior to the stimulation with LPS (500 ng/mL) for 24 h. Afterwards, cells were rinsed twice with saline solution and incubated 1 h at 37 °C with 20 µM DCFH-DA. Then, cells were washed and kept in saline solution for 30 min at 37 °C. Intracellular production of ROS was measured by fluorescence detection of dichlorofluorescein (DCF) as the oxidized product of DCFH-DA on a spectrophotometer plate reader (495 nm excitation and 527 nm emission).

#### 4.6.4. NO Determination

The NO concentration in the culture media was established by measuring nitrite formed by the oxidation of NO, using the Griess reagent kit, according to manufacturer instructions. The detection limit of this method is 1 µM. Briefly, microglia cells were seeded in a 12-well plate at a density of 1 × 10^6^ cells per well and pre-incubated with compounds (0.1 and 1 µM) for 1 h and then stimulated with LPS (500 ng/mL) for 24 h. Thereafter, the following were mixed in a microplate: 150 µL of cells supernatant, 130 µL of deionized water, and 20 µL of Griess Reagent, which was incubated for 30 min at room temperature. The absorbance was measured on a spectrophotometer plate reader at a wavelength of 548 nm.

#### 4.6.5. Statistical Analysis

Results were expressed as mean ± SEM of a minimum of three experiments, repeated twice or three times. Comparisons were performed using Student’s *t*-test or one-way ANOVA with Dunnett’s *post hoc* analysis. *p* values < 0.05 were considered statistically significant.

## Figures and Tables

**Table 1:** ^1^H and ^13^C NMR data in ppm for compounds **1** and **2** in CDCl_3_ (500 MHz for ^1^H NMR and 125 MHz for ^13^C NMR data).

	1		2	
No.	*δ*_H_, mult. (*J* in Hz)	*δ*_C_	*δ*_H_, mult. (*J* in Hz)	*δ*_C_
**1**	3.24, t (7.0)	45.3	3.24, t (7.5)	45.5
	3.19, d (7.0)		3.20, d (7.0)	
**2**	4.58, br d (6.5)	75.6	4.59, d (7.0)	75.7
**3**	4.62, br t (3.0)	72.5	4.63, t (3.0)	72.6
**4**	2.44, br sext (5.5)	26.0	2.43, br sext (6.0)	26.1
**5**	1.92, dd (12.0, 6.0)	40.3	1.95, dd (12.5, 6.0)	40.4
	1.36, t (12.5)		1.37, t (13.0)	
**6**	-	90.1	-	90.2
**7**	1.88, dd (12.5, 4.5)	29.8	1.90, dd (12.5, 4.5)	29.9
	1.80, dt (12.5, 3.5)		1.80, dt (12.5, 3.5)	
**8**	1.66, td (13.5, 3.5)	23.7	1.67, td (14.0, 3.5)	23.8
	1.57, dt (13.5, 4.0)		1.57, dt (14.0, 4.0)	
**9**	-	40.0	-	40.5
**10**	-	100.9	-	101.0
**11**	2.08, d (13.0)	41.8	2.11, d (13.0)	42.0
	1.94, d (13.0)		1.93, d (13.0)	
**12**	-	39.9	-	39.8
**13**	2.20, td (12.0, 4.5)	53.1	2.41, td (12.0, 4.5)	48.1
**14**	2.26, br s	32.0	2.24, br s	30.7
	2.24, br s		2.22, br s	
**15**	-	160.0	-	160.1
**16**	5.90, s	125.6	5.92, s	127.0
**17**	-	198.5	-	197.3
**18**	2.69, td (12.0, 6.5)	46.4	2.66, dd (12.5, 6.5)	48.2
**19**	2.62, dd (14.5, 6.5)	42.4	3.02, dq (7.0, 6.5)	45.9
	2.50, dd (14.5, 12.0)			
**20**	-	209.0	-	212.2
**21**	2.83, s	59.1	3.23, s	53.9
**22**	-	36.5	-	40.3
**23**	3.65, d (20.0)	35.9	3.68, d (20.0)	36.1
	2.36, d (20.0)		2.37, d (20.0)	
**24**	-	172.3	-	172.4
**25**	0.99, s	21.1	0.98, s	20.8
**26**	-	-	1.17, d (7.0)	13.9
**27**	2.00, s	24.4	2.01, s	24.6
**28**	0.97, s	18.5	0.99, s	18.5
**29**	1.15, s	18.4	1.21, s	18.4
**30**	0.87, d (7.0)	16.3	0.89, d (7.0)	16.4
**Ac**	-	171.2	-	171.4
	2.11, s	21.1	2.14, s	21.2

## Supplementary Materials

The following are available online at http://www.mdpi.com/1660-3397/16/7/242/s1: HRMS and NMR data for compounds **1** and **2**.
