# Supplementary material for: Zoanthamine Alkaloids from the Zoantharian Zoanthus cf. pulchellus and Their Effects in Neuroinflammation
Source: Mar Drugs. 2018 Jul 20;16(7):242. doi: 10.3390/md16070242 (PMC6071026; doi:10.3390/md16070242)
Supplement: Supplementary file 1 [file marinedrugs-16-00242-s001.pdf]

Supplementary Information for

Zoanthamine alkaloids from the Zoantharian *Zoanthus* cf. *pulchellus* and their effect in neuroinflammation

Paul O. Guillen [1,2], Sandra Gegunde [3], Karla B. Jaramillo [1,4], Amparo Alfonso [3], Kevin Calabro [2], Eva Alonso [3], Jenny Rodriguez [1], Luis M. Botana [3,*], and Olivier P. Thomas [2,*]

[1] ESPOL Escuela Superior Politécnica del Litoral, ESPOL, Centro Nacional de Acuacultura e Investigaciones Marinas, Campus Gustavo Galindo km. 30.5 vía Perimetral, P.O.Box 09-01-5863, Guayaquil, Ecuador.; P.GUILLENMENA1@nuigalway.ie, K.JARAMILLOAGUILAR1@nuigalway.ie, jenrodri@espol.edu.ec

[2] Marine Biodiscovery, School of Chemistry and Ryan Institute, National University of Ireland Galway (NUI Galway), University Road, H91 TK33 Galway, Ireland; kevin.calabro@nuigalway.ie, olivier.thomas@nuigalway.ie

[3] Departamento de Farmacología, Facultad de Veterinaria, Universidade de Santiago de Compostela, 27002 Lugo, Spain; sandra.gegunde@rai.usc.es, amparo.alfonso@usc.es, eva.alonso@usc.es, luis.botana@usc.es

[4] Zoology, School of Natural Sciences and Ryan Institute, National University of Ireland Galway (NUI Galway), University Road, H91 TK33 Galway, Ireland

* Correspondence: luis.botana@usc.es and olivier.thomas@nuigalway.ie; Tel.: +353-91-493563

P **Figure S1**. (+)-HRESIMS analysis of **1**

P **Figure S2**. ^{1}H NMR spectrum of **1** at 500 MHz in $CDCl_3$

P **Figure S3**. COSY NMR spectrum of **1** at 500 MHz in $CDCl_3$

P **Figure S4**. ^{13}C NMR spectrum of **1** at 125 MHz in $CDCl_3$

P **Figure S5**. HSQC NMR spectrum of **1** at 500MHz in $CDCl_3$

P **Figure S6**. HMBC NMR spectrum of **1** at 500MHz in $CDCl_3$

P **Figure S7**. HMBC NMR spectrum of **1** at 500MHz in $CDCl_3$

P **Figure S8**. (+)-HRESIMS analysis of **2**

P **Figure S9**. ^{1}H NMR spectrum of **2** at 500 MHz in $CDCl_3$

P **Figure S10**. COSY NMR spectrum of **2** at 500 MHz in $CDCl_3$

P **Figure S11**. ^{13}C NMR spectrum of **2** at 125 MHz in $CDCl_3$

P **Figure S12**. HSQC NMR spectrum of **2** at 500 MHz in $CDCl_3$

P **Figure S13**. HMBC NMR spectrum of **2** at 500 MHz in $CDCl_3$

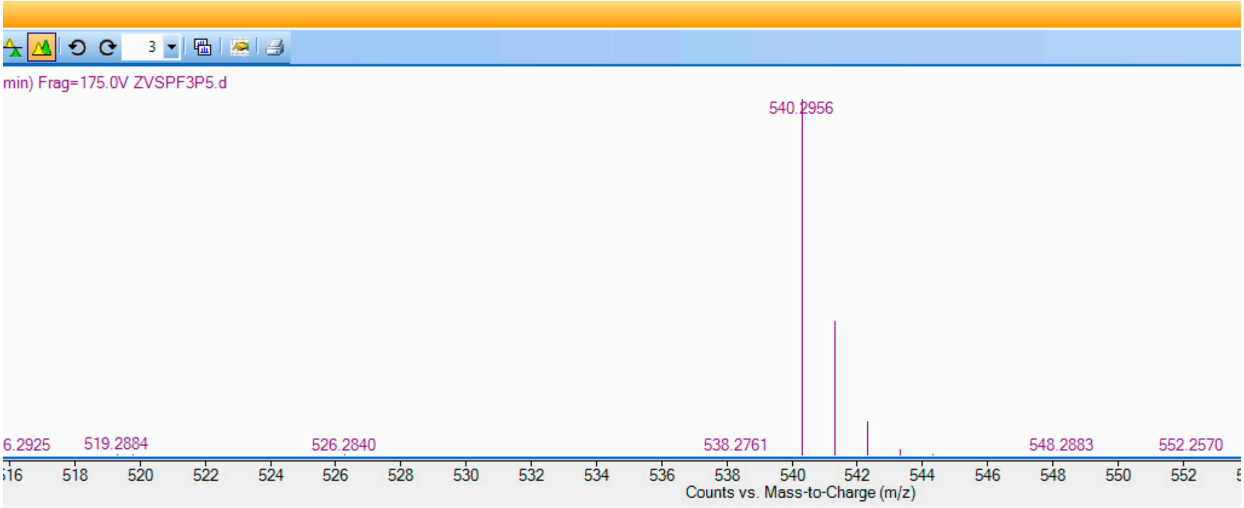

Figure S1. (+)-HRESIMS analysis of **1**.

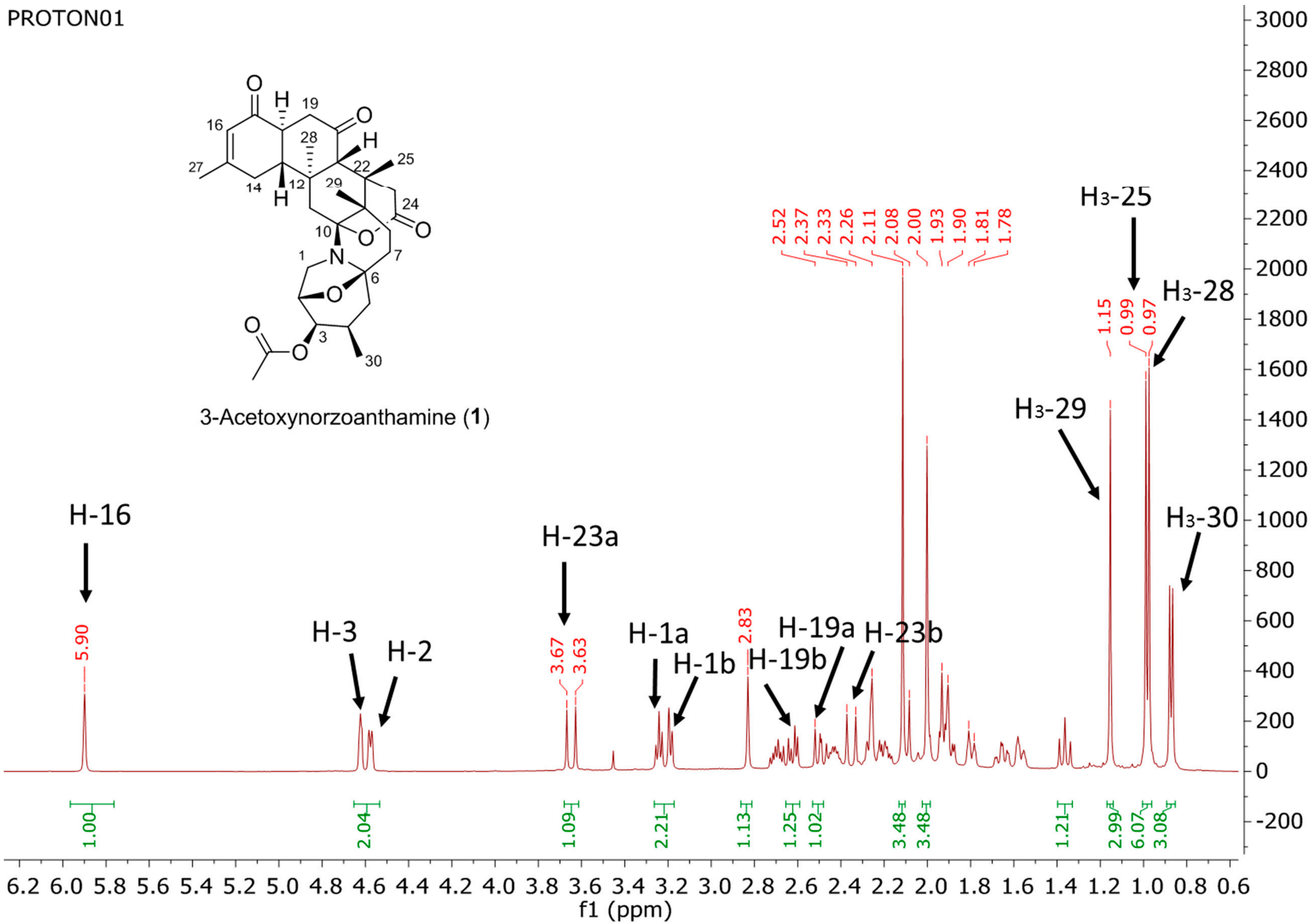

Figure S2. ^{1}H NMR spectrum of **1** at 500 MHz in $CDCl_3$

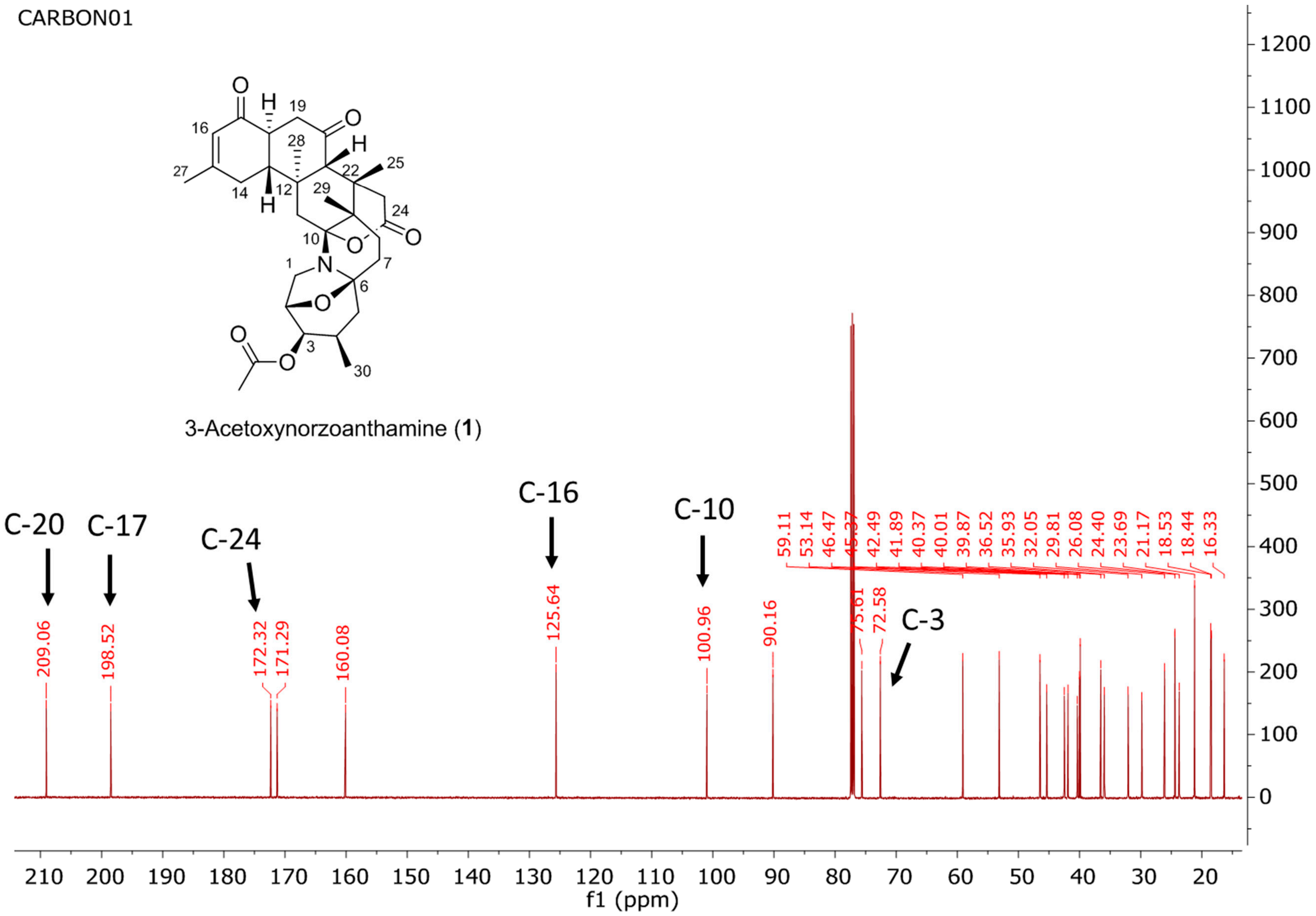

Figure S3. ^{13}C NMR spectrum of **1** at 125 MHz in $CDCl_3$

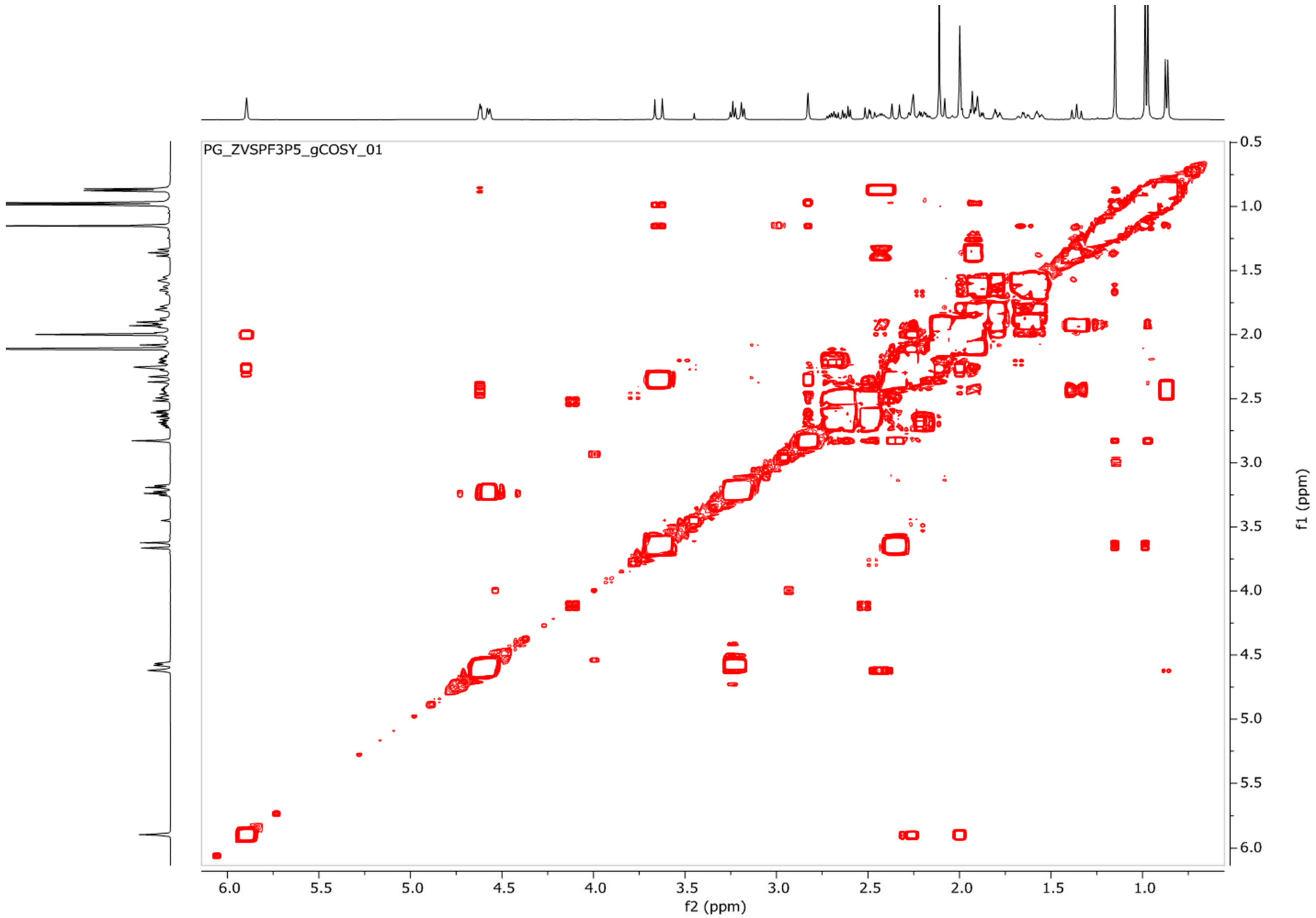

Figure S4. COSY NMR spectrum of **1** at 500 MHz in $CDCl_3$

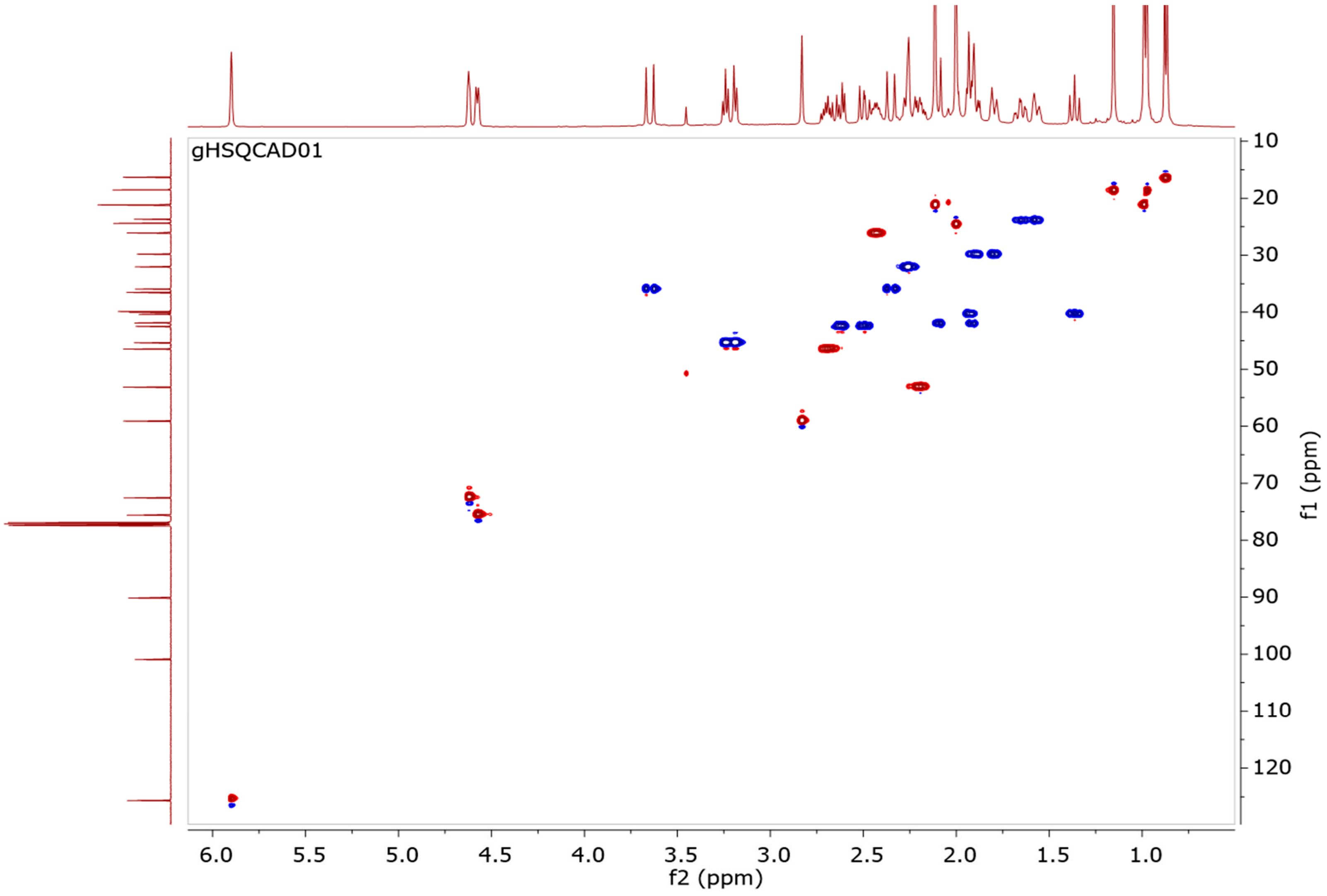

Figure S3. HSQC NMR spectrum of **1** at 500MHz in $CDCl_3$

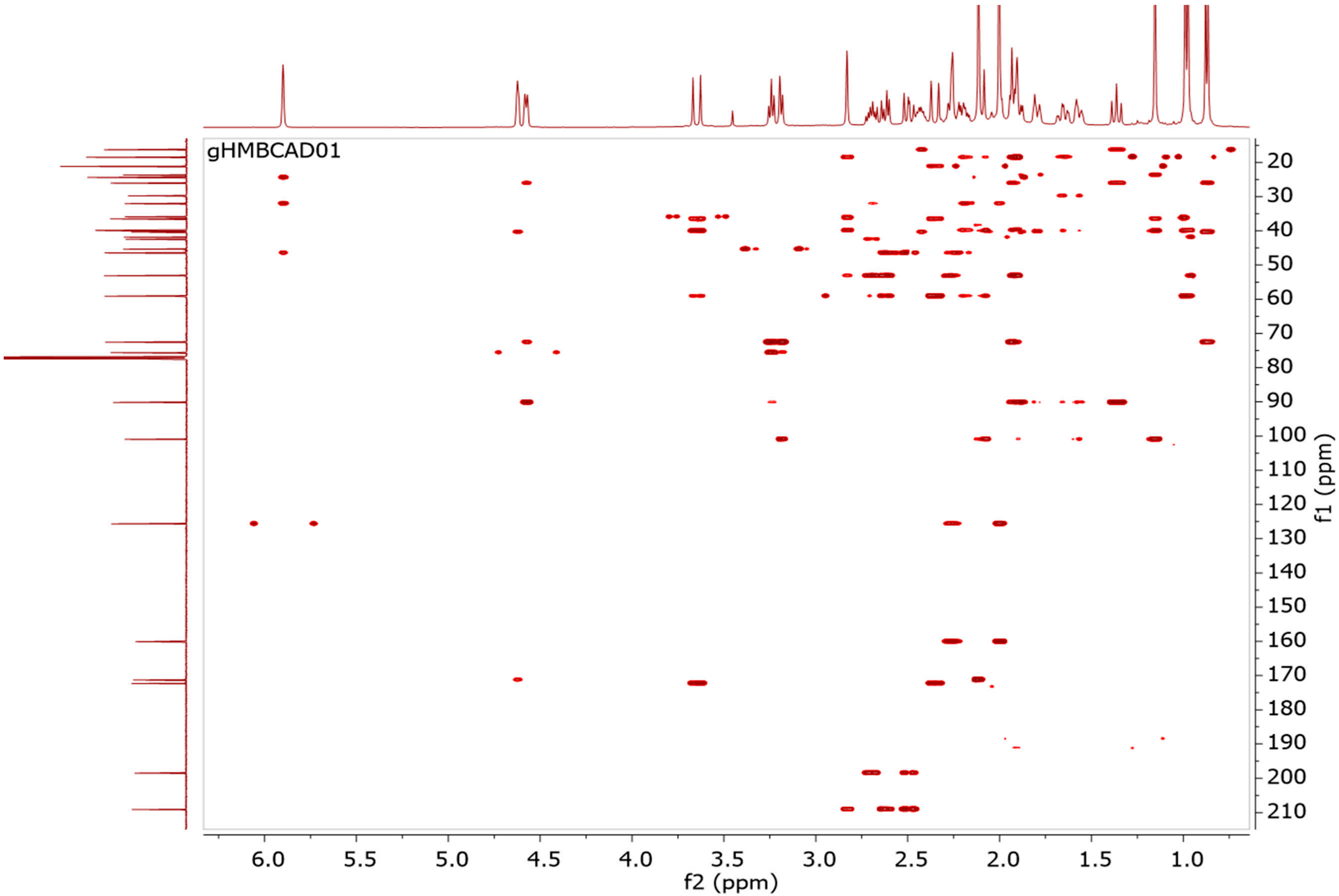

Figure S4. HMBC NMR spectrum of **1** at 500MHz in $CDCl_3$

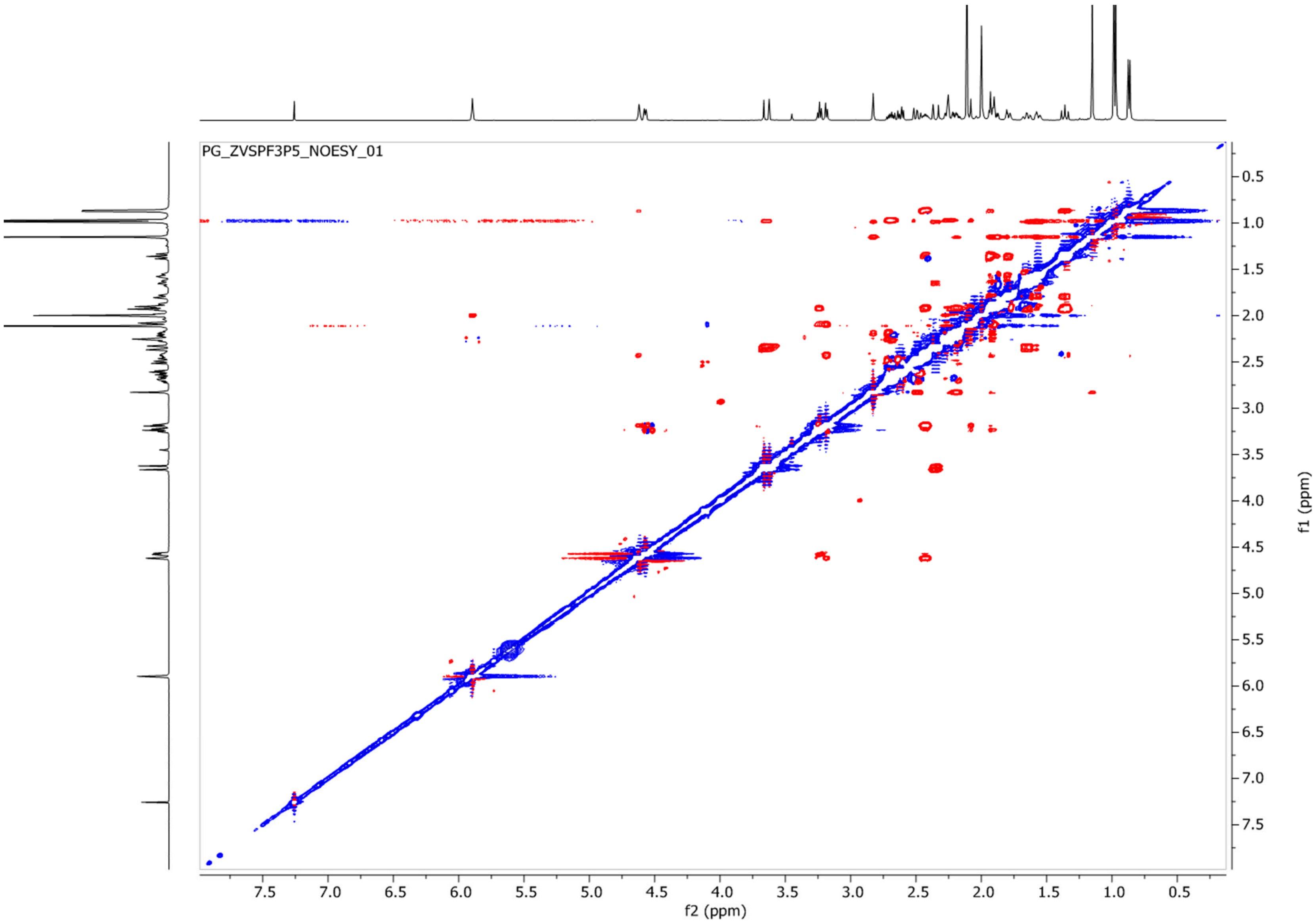

Figure S7. NOESY NMR spectrum of **1** at 500 MHz in $CDCl_3$

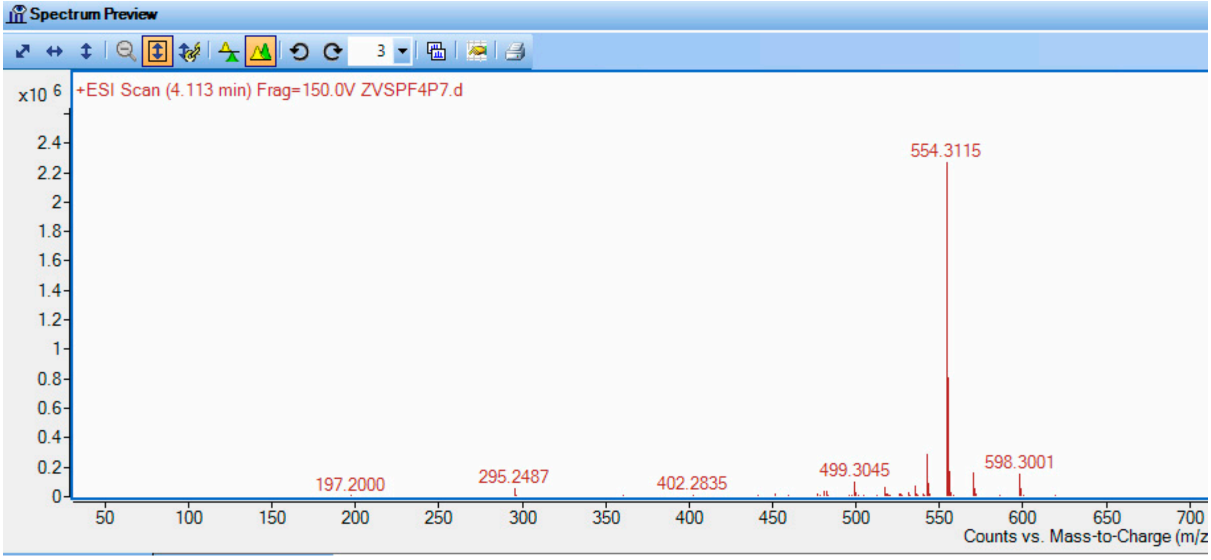

Figure S8. UHPLC-qToF analysis of **2** in (+)-ESI

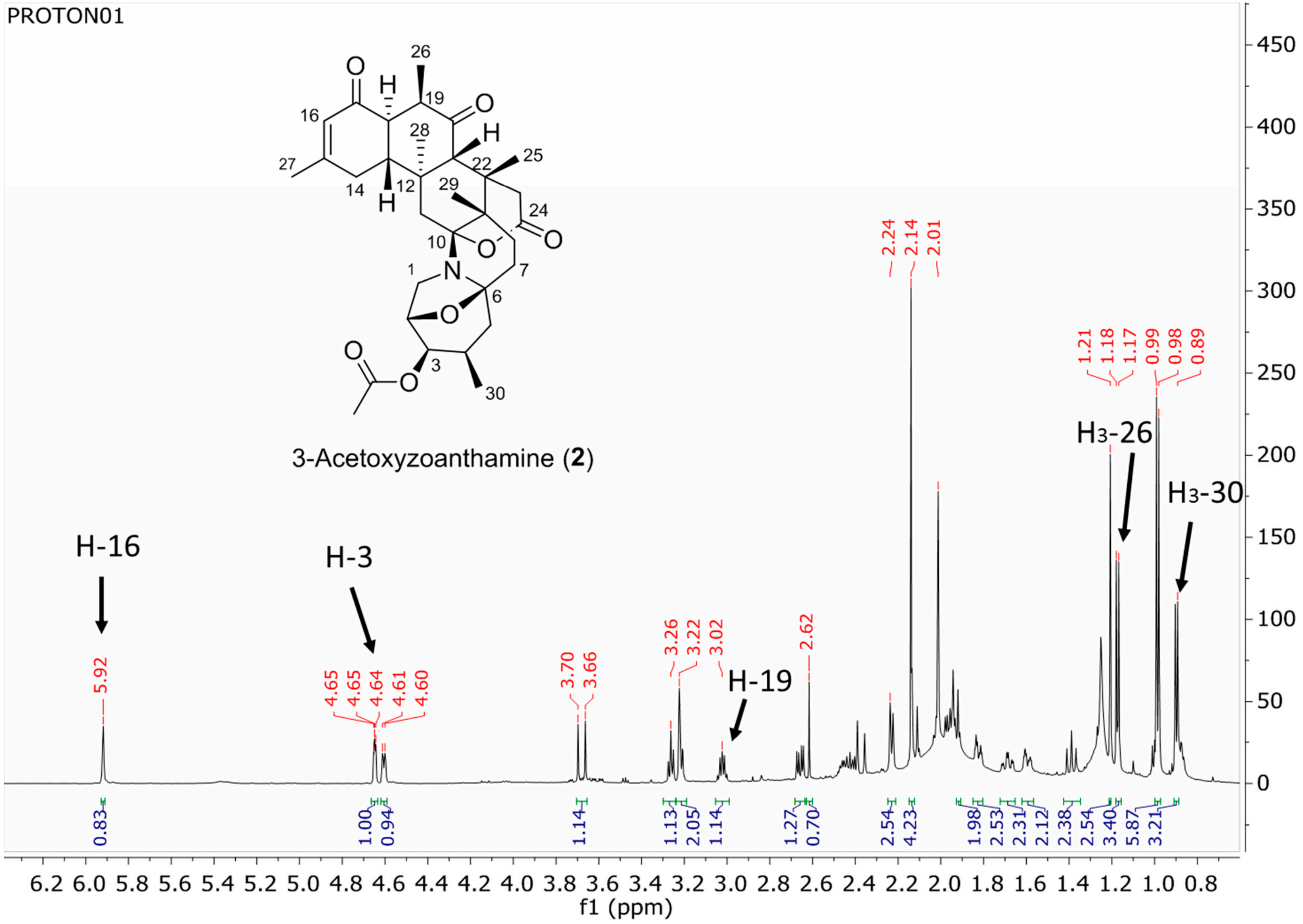

Figure S5. ^{1}H NMR spectrum of **2** at 500 MHz in $CDCl_3$

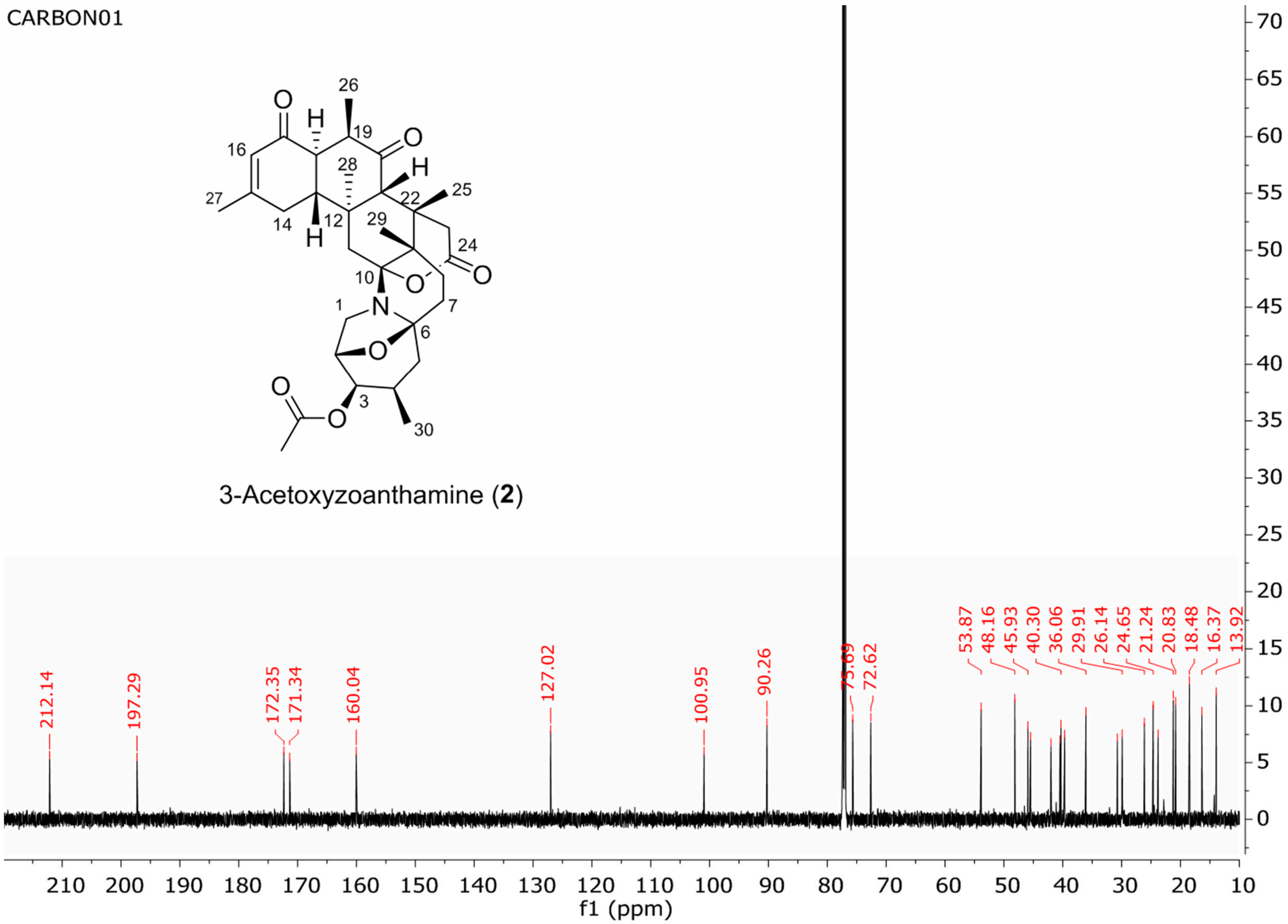

Figure S9. ^{13}C NMR spectrum of **2** at 125 MHz in $CDCl_3$

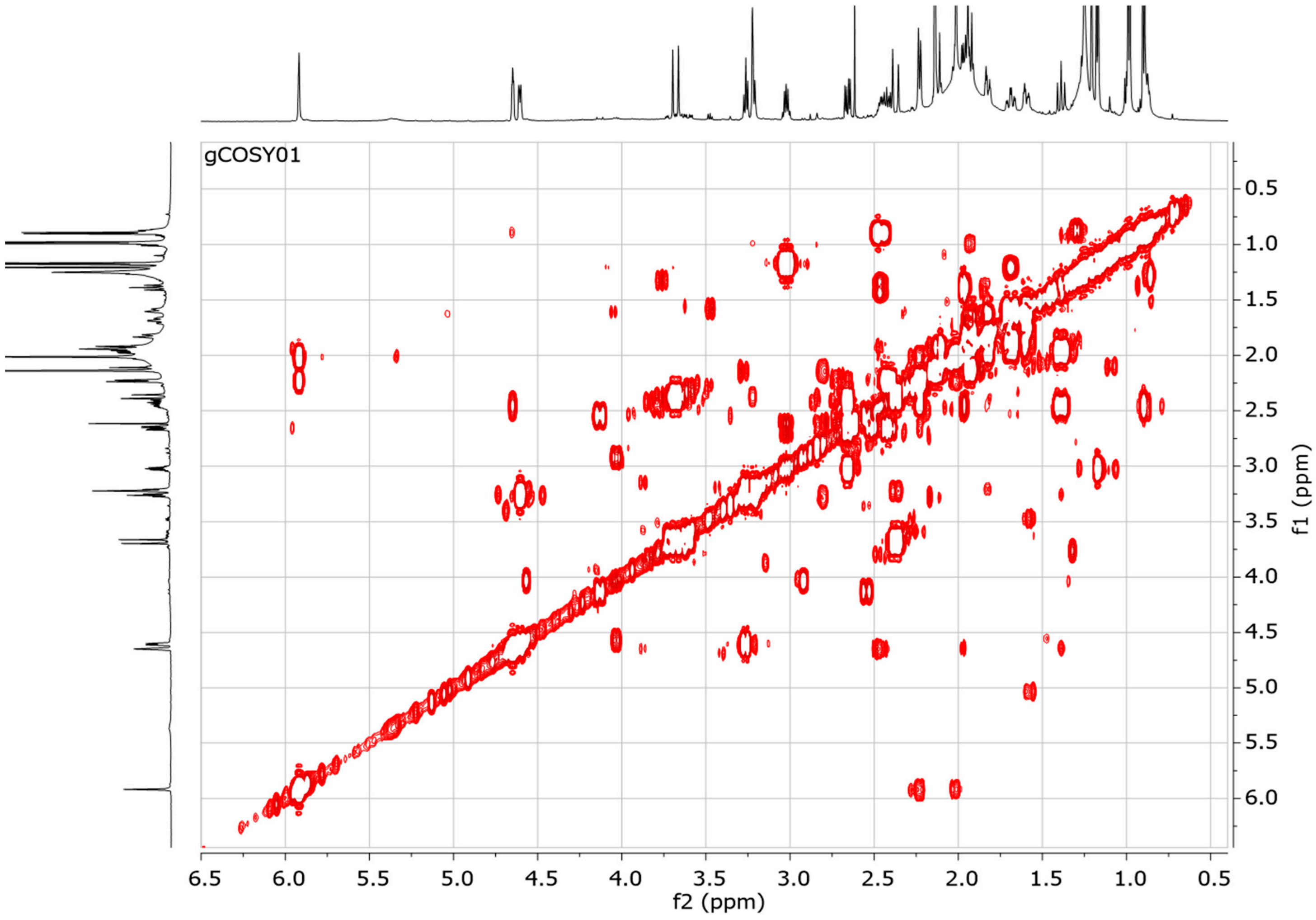

Figure S10. COSY NMR spectrum of **2** at 500 MHz in $CDCl_3$

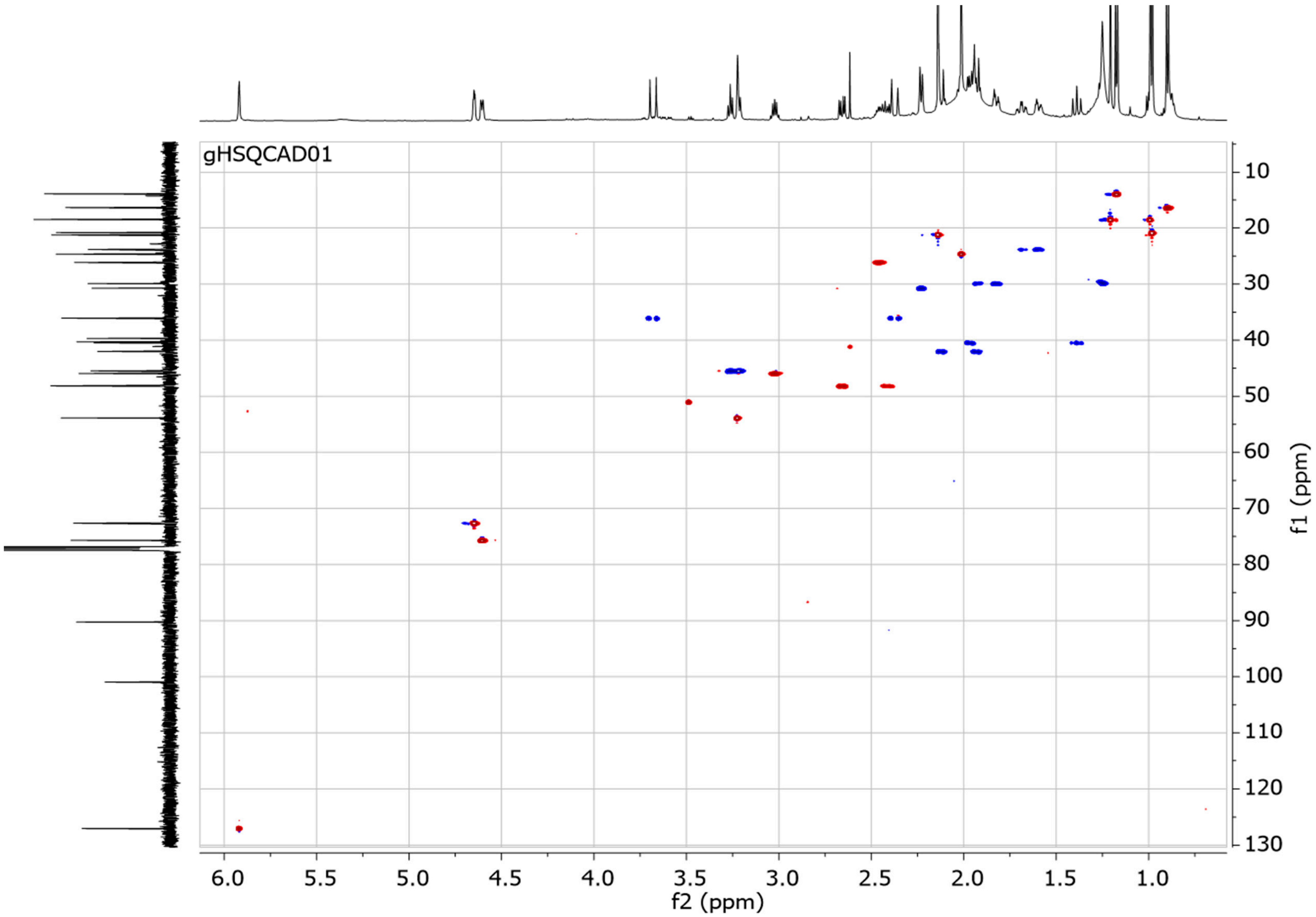

Figure S6. HSQC NMR spectrum of **2** at 500 MHz in $CDCl_3$

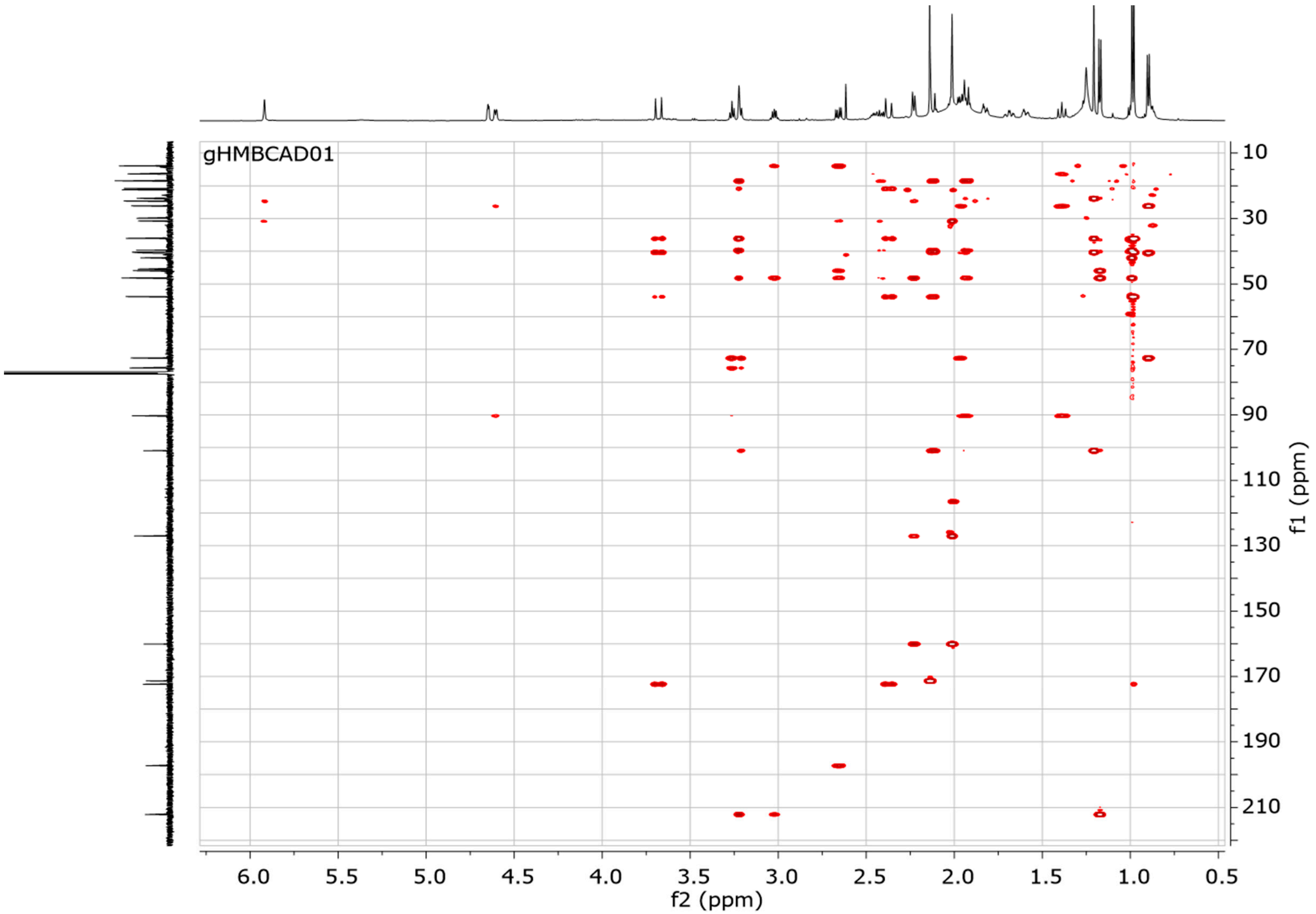

Figure S7. HMBC NMR spectrum of **2** at 500 MHz in $CDCl_3$
